# Edge magnetism of Heisenberg model on honeycomb lattice

**DOI:** 10.1038/srep43678

**Published:** 2017-03-07

**Authors:** Wen-Min Huang, Toshiya Hikihara, Yen-Chen Lee, Hsiu-Hau Lin

**Affiliations:** 1 Department of physics, National Chung Hsing University, Taichung 40227, Taiwan; 2 Faculty of Science and Technology, Gunma University, Kiryu, Gunma 376-8515, Japan; 3 Department of Physics, National Tsing Hua University, Hsinchu 30013, Taiwan

## Abstract

Edge magnetism in graphene sparks intense theoretical and experimental interests. In the previous study, we demonstrated the existence of collective excitations at the zigzag edge of the honeycomb lattice with long-ranged Néel order. By employing the Schwinger-boson approach, we show that the edge magnons remain robust even when the long-ranged order is destroyed by spin fluctuations. Furthermore, in the effective field-theory limit, the dynamics of the edge magnon is captured by the one-dimensional relativistic Klein-Gordon equation. It is intriguing that the boundary field theory for the edge magnon is tied up with its bulk counterpart. By performing density-matrix renormalization group calculations, we show that the robustness may be attributed to the closeness between the ground state and the Néel state. The existence of edge magnon is not limited to the honeycomb structure, as demonstrated in the rotated-square lattice with zigzag edges as well. The universal behavior indicates that the edge magnons may attribute to the uncompensated edges and can be detected in many two-dimensional materials.

Exchange interactions between local magnetic moments, often described by the Heisenberg model and its derivatives, lead to rich and sometimes exotic phases in quantum magnetism^1^. For instance, the excitation gap in the integer-spin chain proposed by Haldane^2^ stimulated theoretical investigations and was later verified in experiments^3^,^4^. Antiferromagnetism in two-dimensional square lattice has been studied extensively because of its adjacency to unconventional superconductivity in cuprates^5^ and iron-based materials^6^. Recent breakthrough shows that the *S* = 1/2 Heisenberg model on Kagome lattice exhibits exotic spin-liquid ground state^7^,^8^,^9^,^10^,^11^,^12^,^13^,^14^,^15^ due to strong frustrations. Moreover, it has been demonstrated that superexchange interactions between ultracold atoms can be realized in optical lattices^16^. It may provide a different route to understand various ground states of the Heisenberg model on different lattice structures.

It is known that boundary effects give rise to fractionalized excitations in integer-spin chains^17^,^18^ but are less studied for spin systems in higher dimensions. The importance to understand the boundary effects in the Heisenberg model is echoed by plausible edge magnetism in graphene nanoribbons^19^,^20^,^21^,^22^,^23^,^24^,^25^,^26^,^27^,^28^,^29^,^30^. By bottom-up approaches, graphene materials with atomic-sharp zigzag edges have been fabricated successfully. Experimental observations verify the existence of the edge-localized states^31^,^32^,^33^ with strong electronic correlations^34^. On the zigzag edges of narrow graphene nanoribbons, magnetic order has been spotted at room temperature^35^. Skipping the technical details, the emergence of edge magnetism can be understood by Lieb’s ferrimagnetism, i.e. the local mismatch of sublattice sites. Monte Carlo simulations^36^ demonstrated mean-field like ferromagnetic moment near the zigzag edges of graphene nanoribbons. Unlike the usual ferromagnetic magnons with quadratic dispersion, these collective excitations near the zigzag edges exhibit linear dispersion. Ignoring the quantum fluctuations momentarily, recent spin-wave calculations^37^ for the Heisenberg model on honeycomb lattice show that the dispersion of the ferromagnetic edge magnon is indeed linear.

Despite of fruitful theoretical progress in the past few years, it remains puzzling how the ferromagnetic edge magnon acquires robust linear dispersion in the presence of charge and spin fluctuations. The absence of quadratic dispersion hints that the boundary field theory for the edge magnon must tie up with its bulk counterpart. In this report, we study edge magnetism of the Heisenberg model on honeycomb nanoribbon with zigzag edges as shown in Fig. 1. We employ the Schwinger-boson approach to compute the dispersion of the edge magnons. It is quite interesting that the linear dispersion remains robust even when the long-ranged Néel order is destroyed by the spin fluctuations. In the field-theory limit, the dynamics of the edge magnons is captured by the one-dimensional Klein-Gordon (K-G) equation. The boundary conditions give rise to evanescent modes propagating along the edge with imaginary momentum in the transverse direction. It is quite fascinating that the parameters characterizing the edge magnons are directly related to those in the bulk. The existence of the edge magnons is not limited to the honeycomb structure, as demonstrated in the rotated-square lattice with zigzag edges as well. The universal behavior indicates that the emergence of edge magnons is directly related to the uncompensated edges and can be detected in many two-dimensional materials.

Our derivations provide natural explanation for the linear dispersion and reveal the connection between the boundary field theory for edge magnons and its bulk counterpart. To further clear up the role of the long-ranged Néel order, we also perform density-matrix renormalization group (DMRG) calculations in graphene nanoribbons. Our previous DMRG studies^22^ has demonstrated the presence of edge magnetism in graphene nanoribbons even though quantum fluctuations destroy the long-ranged order. Why can the collective excitations survive on the edge even though the long-ranged order is already destroyed? The question remains open at this point. But, our DMRG calculations demonstrate that the Néel state is very close to the ground state and provide an indirect hint why the collective excitations can survive even though the long-ranged order is gone.

## Results

### Heinsenberg model

To explore the boundary effects for nanoribbons with honeycomb structure, we first write down the Heisenberg Hamiltonian for the exchange interactions,


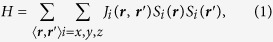


where 〈***r**, **r***′〉 denotes all nearest-neighbor pairs with ***r*** = (*x, y*) on the honeycomb lattice. The exchange couplings are *J*_*x*_ = *J*_*y*_ = *J* and *J*_*z*_ = *γJ* with anisotropy *γ* ≥ 1. Given the on-site interaction *U* and the nearest-neighbor hopping amplitude *t* in graphene, they lead to the exchange coupling *J* = 4*t*^2^/*U* > 0 in the strong-interaction limit.

**Figure 1:**
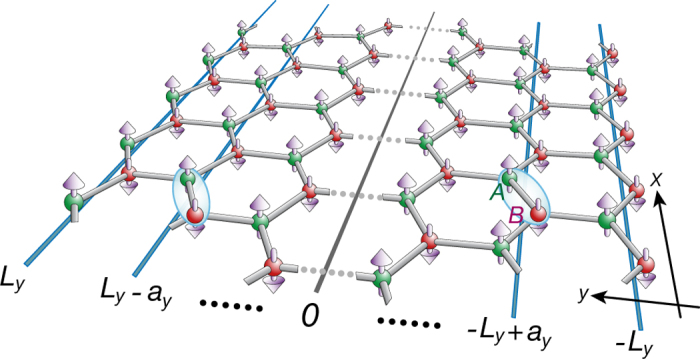
Néel state in a honeycomb nanoribbon with zigzag edges. Spin orientations (purple arrows) on sublattice *A* (green dots) and sublattice *B* (red dots) are opposite to each other. The choice of unit cell is highlighted by the shaded blue circle.

In deriving the effective field theory for the edge magnons, to simplify the algebra, we start with the Holstein-Primakov (HP) bosons in the presence of the Néel order as shown in Fig. 1. Neglecting the interactions between the HP bosons, the effective Hamiltonian within the spin-wave approximation is


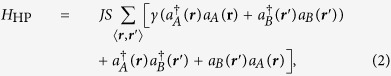


where *S* is the magnitude of the spin and *a*_*A*/*B*_ are annihilation operators for the HP bosons on sublattices *A*/*B*. Since the spin-wave Hamiltonian is bilinear, it is straightforward to write down its equivalent equations of motion in first-quantization language. Following the same steps developed in ref. 37, the dynamics is described by the coupled Harper equations,


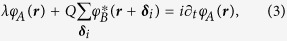



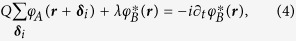


where ***r*** denotes the lattice sites for honeycomb lattice and ***δ***_*i*_ are the vectors pointing to the nearest neighbors. The wave functions on different sublattices are 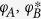, where the conjugation arises from the opposite spin orientation. The key parameters are the hopping amplitude *Q* = *JS* and the chemical potential *λ* = *zγQ*, where *z* is the number of nearest neighbors. It is important to emphasize that the magnon carries quantum number 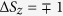 and sets the normalization condition,


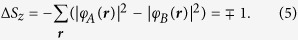


The above Harper equations can be solved exactly, delivering a single-branch ferromagnetic magnon near the zigzag edge with linear dispersion. In the following, we would like to develop general field-theory descriptions to explicitly reveal the connection between magnons in the bulk and those at the edge.

### Field theory in the bulk

In the field-theory limit, we introduce the smooth-varying fields, 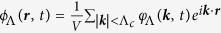, where the momentum summation is restricted to the vicinity of ***k*** = 0 with a cutoff Λ_*c*_. For these smooth-varying fields, spatial variable ***r*** can be treated as continuous and no longer restricted to the lattice sites. In consequence, spatial derivatives are well-defined. Making use of the displacement operator, 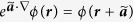, the Harper equations can be represented in the matrix form,


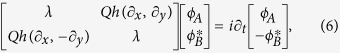


where 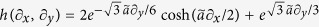 for the honeycomb lattice, and 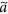 being the lattice constant. Keeping the lowest order in the gradient expansions and eliminating the field 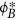, the dynamical equation solely for the field *ϕ*_*A*_ can be derived. It is not surprising that the effective field theory turns out to be the well-known Klein-Gordon equation in two dimensions,


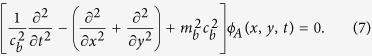


The spin-wave velocity in the bulk is 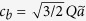 and the effective mass is 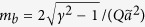. One can also eliminate the field *ϕ*_*A*_ and show that 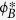 also satisfies the same K-G equation. These results are not surprising because antiferromagnet in the low-energy limit is relativistic. Without the annoying spin kinematics, spin operators can be viewed as canonical bosons and K-G equation becomes a natural description.

It is important to keep in mind that *ϕ*_*A*_ and 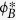 are antiparticles to each other. As required by relativity, they always appear in pairs and explain the double degeneracy for magnons in an antiferromagnet. Furthermore, when the anisotropy disappears, *γ* = 1, the excitation gap for the magnon 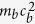 also disappears as expected from the Goldstone’s theorem.

### Field theory at the edge

One can also introduce the smooth-varying fields on the edge and applies the same techniques to derive the boundary field theory. Since our goal is to demonstrate the connection between the field theories in the bulk and at the edge, it is wise to write down the field-theory presentation for the boundary conditions. For the honeycomb nanoribbon considered here, at the upper edge (*y* = *L*_*y*_) where the outmost sites belong to sublattice *A*, the boundary condition gives the constraint 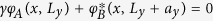. On the other hand, for the lower edge (*y* = −*L*_*y*_), the outmost sites belong to sublattice *B* and the boundary condition leads to 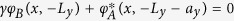. As long as the transverse width *L*_*y*_ is finite, edge magnons on opposite edges entangle together and complicate the problem. For simplicity, let us temporarily assume that the transverse width *L*_*y*_ is sufficiently large so that the coherent overlap between opposite edges can be ignored.

Eliminating the field 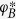 with the help of Eq. (6), the boundary conditions on the upper edge is simplified to the constraint on the field *φ*_*A*_ solely,


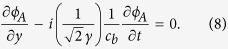


Note that the above relation impose constraint on how the (imaginary) momentum in the transverse direction renormalizes the propagation of magnons on the upper edge. It can be shown that edge magnons on the upper boundary carry quantum number Δ*S*_*z*_ = −1 with evanescent wave function 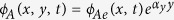, where *α*_*y*_ > 0 is the imaginary momentum along the transverse direction. Substituting the boundary constraint into the bulk K-G equation, the dimensionality is effectively reduced to one. The resultant equation for the edge magnon is the one-dimensional K-G equation,


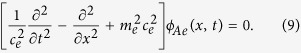


The spin-wave velocity and the effective mass for the edge magnon are related to its bulk values,


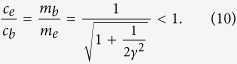


**Figure 2:**
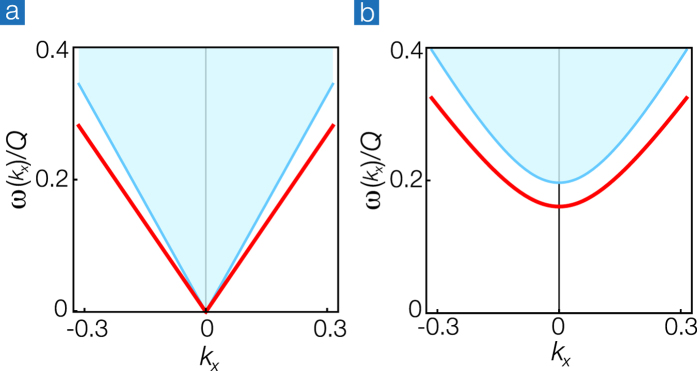
Dispersions for edge and bulk magnons. (**a**) In isotropic limit *γ* = 1 and (**b**) with slight anisotropy *γ* = 1.01. The dispersion of edge magnon (red line) in the isotropic case shows linear dependence. The shaded light blue regime represents the continuum of magnons in the bulk.

The above results are plotted in Fig. 2. Since the excitation gap 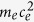 and the spin-wave velocity *c*_*e*_ are smaller, the dispersion for the edge magnon lies below the continuum and remains sharp even when the interactions between magnons are included perturbatively.

### Symmetry argument between two edges

On the lower boundary, the edge magnons carry quantum number Δ*S*_*z*_ = 1 with wave function 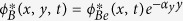. Following similar calculations, one can show that the edge magnon also satisfies the one-dimensional K-G equation with identical parameters. The similarity between the edge magnons on opposite edges calls for a *Z*_2_ symmetry argument. It turns out the discrete symmetry relating these evanescent modes is the parity symmetry *P*_*y*_ in the transverse direction.

Because the Néel state consists of the staggered spin configurations, the operation of parity symmetry needs extra caution. When reversing the *y*-axis, it is clear that the lattice coordinates transform as (*y, A*) → (−*y, B*) and (*y, B*) → (−*y, A*). However, due to the staggered spin configuration in the Néel state, the parity transformation also reverses the spin orientations and causes “charge conjugation” effectively. That is to say, the parity transformation turns particle-like excitations into the hole-like and vice versa. Therefore, the solution under *P*_*y*_ transformation takes the form,


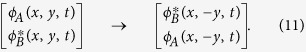


The above symmetry is exactly what happens in the boundary field theory for edge magnons. The boundary field theory supplemented with the symmetry argument fully answers our puzzle. The ferromagnetic magnons satisfies the one-dimensional K-G equation originated from its bulk counterpart with explicit relations. The edge magnons running on upper and lower boundaries carry opposite quantum numbers and are antiparticles to each other related by the *P*_*y*_ parity symmetry. In fact, the whole field theory (including the bulk and the two edges) is fully relativistic and excitations always appear in pairs as required. The confusion mainly arises from the asymmetry of the spatial wave functions for the edge magnons because their antiparticles locate on the opposite edges. In short, the single-branch *ferromagnetic* edge magnon on one zigzag boundary is indeed an *antiferromagnetic* one with its antiparticle running on the distant opposite boundary. The linear dispersion of the edge magnon (with specific relation to the bulk dispersion) now looks more than natural.

### Schwinger-boson approach

The above derivation can be generalized to the Schwinger bosons where the long-ranged order is absent. Here we introduce the Schwinger-boson operators,


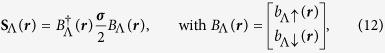


where ***σ*** = (*σ*^*x*^, *σ*^*y*^, *σ*^*z*^) are the Pauli matrices, Λ = *A, B* are the sublattice indices and *b*_↑/↓_ are the annihilation operators of Schwinger bosons with different spin orientations. By rotating the spins on the sublattice *B* along the *y*-axis by the angle *π* (i.e. *S*^*x*^ → −*S*^*x*^, *S*^*z*^ → −*S*^*z*^ and *S*^*y*^ unchanged), the Heisenberg model in Eq. (1) now takes the following form,


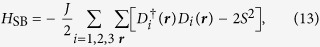


where 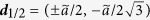, 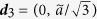 and the pairing operators are defined as *D*_*i*_(***r***) = ∑_*α*_*b*_*Aα*_(***r**)b*_*Bα*_(***r*** + ***d***_*i*_) with *α* = ↑, ↓. Note that the number of the Schwinger bosons, 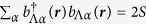, is constrained as usual. Making use of the translational invariance along the *x*-direction, the partial Fourier transformation, 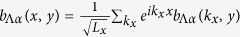 with quantized momenta 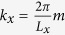, *m* = 1, 2, …, *N*_*x*_, is rather helpful. Within the mean-field approximation, we further decouple the quartic terms and obtain the self-consistent equations,


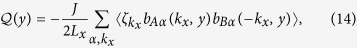



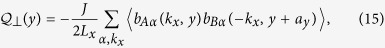



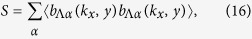


where 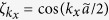 and 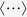 means the ensemble average. It is interesting to compare the Schwinger bosons with the HP bosons at this point: the fluctuations destroy the long-ranged order but only renormalize the effective hopping 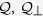 for the spin bosons. This is the underlying reason why the collective excitations at the edge survive even though the long-ranged order is destroyed by quantum fluctuations.

After mean-field decomposition, the effective Hamiltonian for the Schwinger bosons is quadratic,


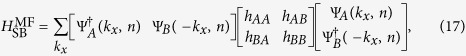


where 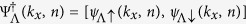 with *n* = *y*/*a*_*y*_, and the matrix elements in the 2 × 2 Hamiltonian matrix are semi-infinite matrices,


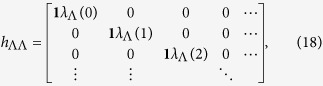



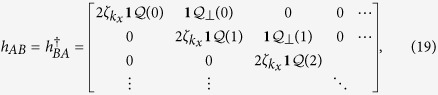


with **1** representing the 2 × 2 identity matrix. Let us consider the semi-infinite graphene along the *y*-direction. The Lagrangian multiplier *λ*_Λ_(*n*) is introduced to enforce the occupancy constraint in Eq. (16), which can be regarded as a local chemical potential of the Schwinger bosons. The mean-field Hamiltonian leads to the Harper equations,


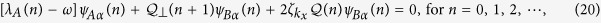



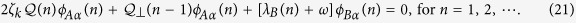


Away from the edge, the homogeneity restores eventually so that we set 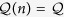, 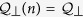 and *λ*_Λ_(*n*) = *λ* to simplify the calculations. Applying generalized Bloch theorem^37^, the solution takes the form of *ψ*_*α*Λ_(*n*) = *ψ*_*α*Λ_(0)*z*^*n*^. The plane-wave solution corresponds to 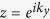 and the dispersion of the Schwinger bosons in the bulk is


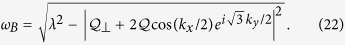


**Figure 3:**
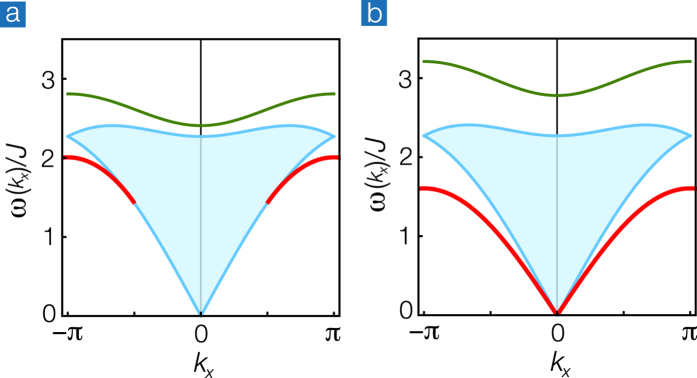
Dispersions of the bulk and the edge Schwinger bosons in a semi-infinite graphene for (**a**) Δ/*J* = 0.5 and (**b**) Δ/*J* = 1 respectively. The blue regime denotes the bulk continuum of the Schwinger bosons, and the red and green lines represent the dispersions of the two edge Schwinger bosons.

For notation simplicity, we have set 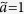 in above. Plugging the dispersion relation into the self-consistent equations, Eqs (14), (15) and (16), the effective hopping and the chemical potential are obtained, 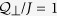, 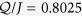 and 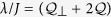 with *k*_*B*_*T*/*J* = 0.01 and *S* = 1/2. The presence of the edge causes non-trivial mixing between counter propagating modes 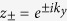, but the spectral continuum of the Schwinger bosons in the bulk remains the same, as shown as the blue regime in Fig. 3.

The emergent ferromagnetic order near the zigzag edge renders the spatial dependence of the parameter *λ*. In principle, it shall be determined self-consistently. However, as long as the ferromagnetic moment at the edge is localized, it is reasonable to assume that only the edge value deviates from its bulk value. That is to say, we can decompose *λ*_*E*_(0) = *λ* + Δ into the bulk value *λ* and the boundary enhancement Δ. Within this approximation, Δ should be treated as a fitting parameter to reproduce the desired ferromagnetic moment at the edge. After some algebra, the boundary conditions read,


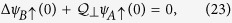



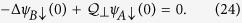


The structure of the effective field theory and the boundary conditions for the Schwinger bosons (without the long-ranged order) take the same form as those for the HP bosons (with Néel order). It is then expected that the edge magnons remain robust except the relevant parameters are renormalized by quantum fluctuations. Together with the Harper equations, the dispersions of the evanescent modes (|*z*| < 1 solutions) for the Schwinger bosons are


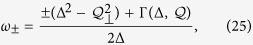


where 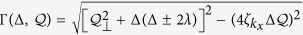. As shown in Fig. 3, the edge magnon gradually develops from the large momenta when the ferromagnetic enhancement is weak Δ/*J* = 0.5 and eventually becomes gapless when the enhancement is large enough Δ/*J* = 1. The discrepancy between the HP-boson approach and the Schwinger-boson approach can be spotted by the extra evanescent mode (green line above the bulk continuum) in Fig. 3. It may arise from the well-known artifact of the Schwinger-boson approach where mode counting often doubles due to the mean-field constraint.

## Discussions

By the Schwinger-boson approach which preserves the *SU*(2) symmetry in the isotropy limit *γ* = 1 explicitly (no long-ranged order), we have shown that the edge magnons survive except the parameters *Q* and *λ* are renormalized due to quantum fluctuations. The robustness of the relativistic boundary field theory is then not a big surprise because the field-theory description for the Schwinger bosons is still relativistic.

**Figure 4:**
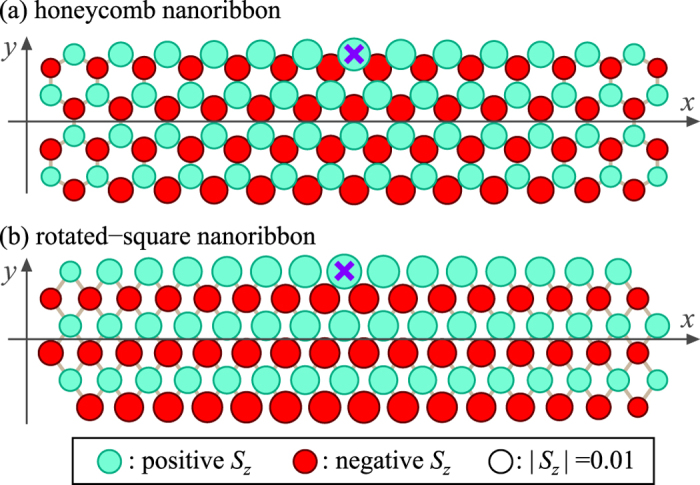
Magnetization profiles by the DMRG calculation. 〈*S*_*z*_(***r***)〉 in (**a**) the honeycomb nanoribbon with zigzag edges and (**b**) the rotated-square nanoribbon. Light (green) and dark (red) circles represent positive and negative values of the spin polarization respectively, while the areas are proportional to the absolute values. Crosses represent the edge site ***r***_0_ for which the local Zeeman field −*hS*_*z*_(***r***_0_) with *h* = 0.01*J* is applied.

To further clear up the role of the long-ranged Néel order, we also perform DMRG calculations in graphene nanoribbons. It is known that the ground state for the Heisenberg model on a finite bipartite lattice is a spin singlet. According to the DMRG calculations, we find that the ground state^30^ is very close to the Néel state. Figure 4 shows the magnetization profiles 〈*S*_*z*_(***r***)〉 in the lowest-energy state of ∑_*r*_*S*_*z*_(***r***) = 0 for the Heisenberg model with a local Zeeman field −*hS*_*z*_(***r***_0_) applied to the center site ***r***_0_ at the upper edge. We show that even a small local field *h* = 0.01 *J* induces a robust Néel order despite of the singlet ground state. The closeness between the spin-singlet ground state and the Néel order may contribute to the robustness of edge magnons in the absence of the long-ranged order.

Note that the edge magnon is not a privilege of the honeycomb nanoribbon with zigzag edges. For the rotated-square nanoribbon as shown in Fig. 4, we repeat the same calculations and find the presence of edge magnons as described in Eq. (9), with different parameters,


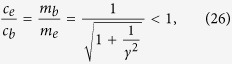


where 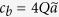 and 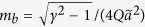. Meanwhile, we also compute the edge magnon on a honeycomb nanoribbon with armchair edges and a square nanoribbon with flat edges. However, in these cases, the fully compensated edges exclude the existence of edge magnons. The results indicate that the emergence of the edge magnon may tie up with the uncompensated edges in the bipartite lattices. However, the proof for general lattice structures and without Néel order is still an open question at the point of writing.

The linear dispersion of the edge magnon in graphene has been speculated in many numerical studies^20^,^27^,^36^, where the Néel order is absent. By extracting the parameters of real materials from these literatures, the spin-wave velocity at the zigzag edge of graphene can be estimated. For reasonable *U* = 2 *eV* and *t* = 2.6 *eV*^27^,^36^, the order of the magnitude is estimated as ~10^6^(*m*/*s*). Most studies neglect the disorder effects so far. In realistic materials, disorder will hybridize the right-moving and left-moving sectors of the magnons and a small gap will be inevitable. However, as long as the disorder is weak, the collective excitations below the continuum can still be found due to the finite energy gap. That is to say, the long-wave length (near *k* = 0) excitations are more sensitive to disorder while the edge magnons with large momenta shall be robust in experimental probes.

Although the Néel order may not play an essential role for the existence of the edge magnons, one shall be cautious to draw similar conclusions for models of graphene nanoribbons with the same geometry. Because edge magnetism in graphene nanoribbons is itinerant in nature, it is not yet clear whether the edge magnon can still be described by the one-dimensional K-G equation derived here. However, recent Monte Carlo simulations^36^ demonstrate the presence of sharp spin-wave excitation with sharp spectral weight, in qualitative agreement with our boundary field-theory description. It would be of vital importance to explore and reveal the true nature of these edge excitations in the future.

## Methods

### Holstein-Primakov bosons

If the Néel order is present, it is convenient to represent the antiferromagnetic Heisenberg model in terms of the Holstein-Primakov bosons,


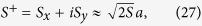



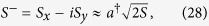



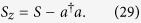


Even though the HP-boson approach is exact, it breaks the SU(2) invariance explicitly and becomes rather awkward when the Néel order is destroyed by quantum fluctuations. Within the spin-wave approximation, we further ignore the interactions between these bosons to reach the quadratic Hamiltonian in Eq. (2).

### DMRG calculation

The magnetization profiles 〈*S*_*z*_(***r***)〉 shown in Fig. 4 are obtained by the finite-system DMRG method^38^,^39^. The number of states kept is up to *χ* = 500. The numerical results of the DMRG method inherently include a systematic error due to the finite cutoff *χ*. To estimate the error, we perform the calculation of 〈*S*_*z*_(***r***)〉 with various *χ* and monitor how the data change with increasing *χ*. For the honeycomb nanoribbon with zigzag edges, we extract the data as a function of the truncation error, which is the sum of the density-matrix weights of discarded states, and then, extrapolate the data to the limit of zero truncation error: Fig. 4 shows the extrapolated values in the DMRG calculations. We note that the truncation error averaged on the final sweep at *χ* = 500 is 8 × 10^−7^ and the differences between the values of 〈*S*_*z*_(***r***)〉 at *χ* = 500 and the extrapolated ones are at most 2.7%, suggesting that the results are accurate enough for our argument. For the rotated-square nanoribbon, the averaged truncation error at *χ* = 500 is 1 × 10^−8^ and the differences between the values of 〈*S*_*z*_(***r***)〉 at *χ* = 500 and 400 are less than 0.074%. It suggests that the convergence of the data with respect to *χ* is achieved sufficiently. We therefore plot the data with *χ* = 500 in Fig. 4.

## Additional Information

**How to cite this article**: Huang, W.-M. *et al*. Edge magnetism of Heisenberg model on honeycomb lattice. *Sci. Rep.* **7**, 43678; doi: 10.1038/srep43678 (2017).

**Publisher's note:** Springer Nature remains neutral with regard to jurisdictional claims in published maps and institutional affiliations.
